# ‘QuickDASH’ to find unique genes and biological processes associated with shoulder osteoarthritis: a prospective case–control study

**DOI:** 10.1186/s13104-024-07035-9

**Published:** 2024-12-19

**Authors:** Samuel J. Lynskey, Stephen D. Gill, Sean L. McGee, Mark Ziemann, Richard S. Page

**Affiliations:** 1 Department of Orthopaedic Surgery, Geelong University Hospital, Geelong, Australia; 2 Barwon Centre for Orthopaedic Research and Education (BCORE), St John of God Hospital, Deakin University, Geelong, Australia; 3 Geelong University Hospital, Geelong, Australia; 4 School of Medicine, Faculty of Health, Deakin University, Waurn Ponds, Geelong, VIC Australia; 5 IMPACT - the Institute for Mental and Physical Health and Clinical Translation, Barwon Health, Deakin University, Geelong, VIC Australia

**Keywords:** Shoulder, Osteoarthritis, Transcriptome, Transcriptomics, Gene expression

## Abstract

**Objective:**

Osteoarthritis (OA) is a disease impacting the synovial joint complex, yet transcriptional changes specific to shoulder OA remain underexplored. This study aims to profile transcriptomic changes in periarticular tissues from patients undergoing shoulder replacement for OA. By correlating these profiles with QuickDASH scores—a validated measure of worsening shoulder function—this research seeks to understand the gene expression changes associated with clinical decline. Capsular tissue biopsies from shoulder OA patients were compared with those from a control group undergoing shoulder stabilization for recurrent instability. This investigation forms part of a larger transcriptomic analysis of painful shoulder conditions which will address the current gap in knowledge regarding the molecular and genetic underpinnings of shoulder OA, rotator cuff tears and cuff-tear arthropathy.

**Results:**

The analysis revealed that genes most strongly associated with increasing QuickDASH scores across tissues were linked to inflammation and stress response. Key pathways involved interleukins, chemokines, complement components, nuclear response factors, and immediate early response genes, reflecting a balance between pro- and anti-inflammatory signalling. Additionally, this study identified unique gene expression patterns in shoulder OA not previously observed in hip and knee OA, along with novel genes implicated in shoulder OA, highlighting areas for future targeted investigation.

*Trial registration* This investigation has been registered with the Australian New Zealand Clinical Trials Registry (ANZCTR), registered on the 26th of March 2018, registration number: 12618000431224, accessible from: https://anzctr.org.au/Trial/Registration/TrialReview.aspx?id=374665&isReview=true

**Supplementary Information:**

The online version contains supplementary material available at 10.1186/s13104-024-07035-9.

## Introduction

Osteoarthritis (OA) is a disease of the synovial joint complex and demonstrates a complex interplay between joint destruction and maladaptive repair [1–4]. The genetic loci associated with hip and knee OA are different, suggesting diverse genetic risk and pathophysiological mechanisms manifest disease within different synovial joints [5]. Transcriptomic datasets are being evaluated for effector genes in hip and knee OA that can be targeted therapeutically [6]. Transcriptomic analyses investigating shoulder OA are limited [7–9]. By investigating transcript-level changes in different periarticular tissues, the molecular pathways of disease that are present in end stage *shoulder* OA may help to understand modifiable processes involved in early stages of disease as a target for alternate therapies.

### Aims

#### Primary aim

To describe gene expression patterns in different periarticular shoulder tissues in patients with advanced OA associated with a patient reported outcome measure (PROM), the QuickDASH score, a marker of worsening pain and disability.

#### Secondary aim

To compare gene expression in osteoarthritic capsular tissue with capsular tissue in patients undergoing arthroscopic stabilisation for recurrent shoulder instability.

## Main text

### Methods

#### Participants and Surgical methods

This is a prospective case–control series of six patients undergoing total shoulder replacement for OA; as compared to twenty-five patients who underwent arthroscopic shoulder stabilisation for recurrent instability, which formed the control group. [10] Eligible patients had experienced more than 6-months of symptoms correlating with characteristic osteoarthritic radiographic changes. Patients with cuff arthropathy according to Hamada [11] underwent reverse total shoulder replacement (one of six patients, patient 3, for Walch B2 morphology, rather than cuff-tear arthropathy), whereas those *without* full thickness rotator cuff tear on ultrasound or MRI underwent anatomic total shoulder replacement. Patients with inflammatory arthropathies; prior shoulder fracture or dislocation; or corticosteroid injection within three months of surgery were excluded. Twenty-five patients who underwent arthroscopic shoulder stabilisation for recurrent instability formed the control group. Eligibility criteria for the control group included MRI evidence of an isolated labral tear without degenerative or inflammatory arthropathy, or rotator cuff tear. Further, patients who had an instability-episode within eight weeks of surgery were excluded.

Surgeries were performed and tissue biopsies obtained per protocol, by three specialised shoulder surgeons, from Bone, Capsule, Fat, Muscle and Synovial tissue in the OA group, and capsule in the control group, *totalling 55 tissue samples for next generation sequencing*.

The detailed methodology regarding our RNA extraction, library preparation, and RNA sequencing methods can be found in our protocol paper [10]**.** In brief, using a handheld homogeniser, ~ 20 mg of tissue was homogenised in trizol and total RNA was isolated using RNeasy columns (Qiagen, Hilden, Germany), according to manufacturer’s instructions. Total RNA was eluted with 40 µL of RNase-free water. Sequencing libraries were generated from 0.5 µg of total RNA using TruSeq Stranded Total RNA preparation kit (Illumina, San Diego, USA) as per manufacturer’s instructions. Genome-wide mRNA levels were measured using the NovaSeq 6000 Sequencing System (Illumina, San Diego, USA).

Fastq files underwent quality trimming with Skewer v0.2.2 to remove bases from the 3’ ends with Phred quality less than 20 [12]. Reads were the mapped to the human transcriptome (Gencode V37) using Kallisto v0.46.2 [13]. Transcript-level counts were read into R v4.2.1 and were aggregated to the gene level for downstream analysis. For differential expression analysis, we used DESeq2 1.36.0 to identify genes whose expression associated with QuickDASH score [14]. This was repeated separately for bone, capsule, fat, muscle and synovial samples. Further, differential gene expression was determined in capsular tissue in OA compared with instability. Multi-contrast pathway analysis with mitch [15] was undertaken referencing Reactome pathways (Downloaded November 2021) [16] to identify differentially expressed pathways that have an association with QuickDASH score across all tissue type; and those differentially expressed in capsular tissue in OA compared with capsular tissue in instability [17]. DESeq2 test statistic of all detected genes were used as an input to mitch to ascertain which Reactomes were positively or negatively associated with QuickDASH scores for all OA tissues. Genes and pathways with a false discovery rate (FDR) < 0.05 were considered significant. Code: https://github.com/markziemann/shoulder-instability-osteroarthritis/blob/main/oa_dge.Rmd. Data discussed in this publication has been deposited in NCBI's Gene Expression Omnibus [18], and accessible through GEO Series accession number GSE281476 (https://www.ncbi.nlm.nih.gov/geo/query/acc.cgi?acc=GSE281476).

#### Clinical outcomes

Disabilities of the Arm, Shoulder, and Hand Questionnaire Short Version (QuickDASH) [19] (Supplementary Table 1), group details and comparative statistics (Supplementary Table 2) were documented.

**Fig. 1:**
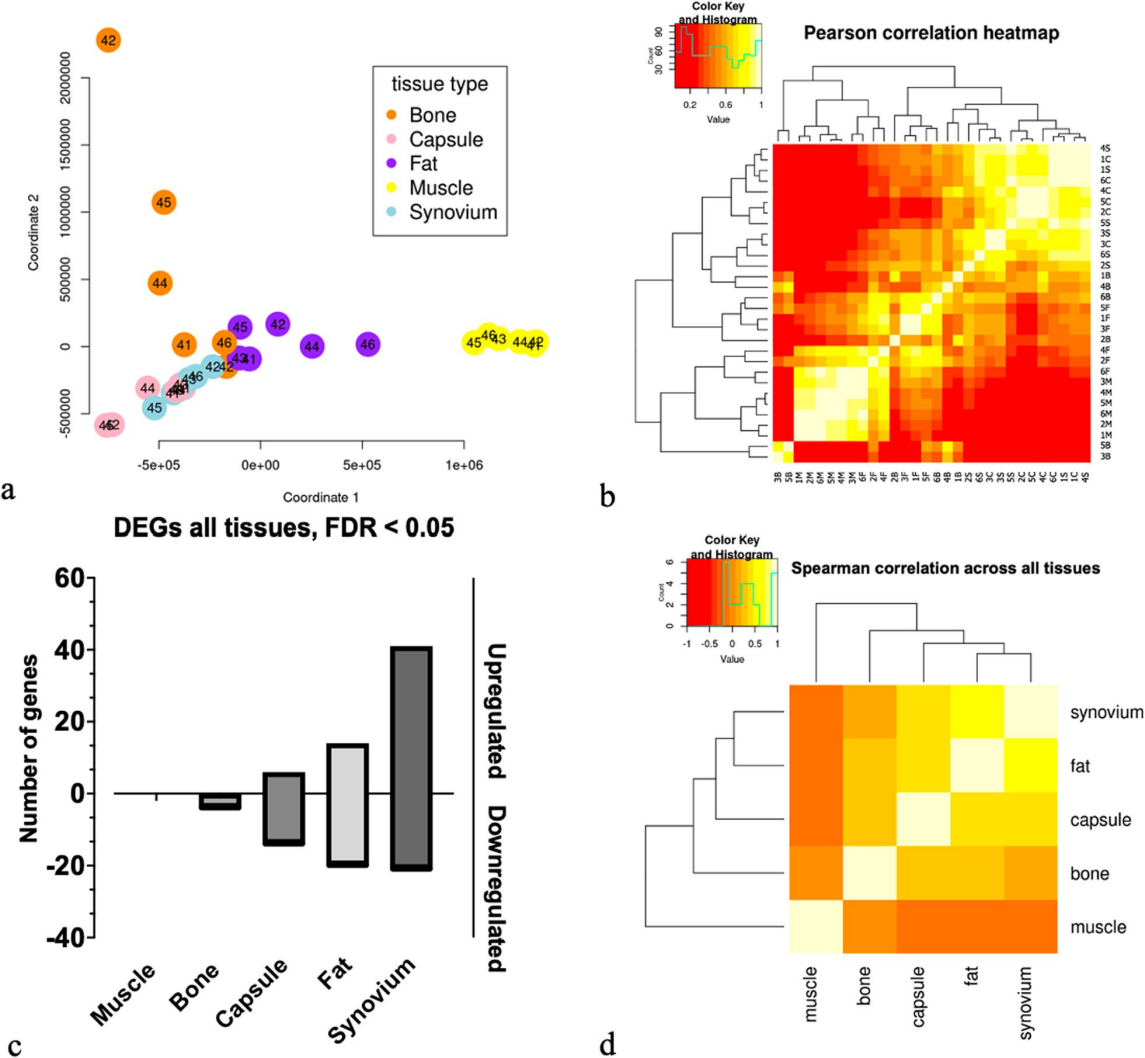
**a** MDS plot to show the variation between tissue samples. Muscle samples to the right, Fat samples in the centre, Capsule samples in the lower left and Synovial samples between Capsule and Fat. However, the Bone samples were less tightly clustered. The result of the MDS is confirmed in the correlation heatmaps (**b**, **d**). **b** Pearson correlation heatmap and colour histogram, Capsule (C), Fat (F), Muscle (M), Synovium (S) and Bone (B) for patients 1 through to 6. Patient 3 underwent RTSR. Figure 1c. Number of DEGs according to tissue type, FDR < 0.05. Figure 1d. Spearman correlation heatmap and colour histogram demonstrating clustering of samples by tissue type; Muscle, Fat, Capsule, Synovium, and Bone

Quality control checks for transcriptomic analysis confirmed that all samples had a minimum of 15 million reads, ensuring robust sequencing depth for downstream analyses (Supplementary Fig. 1). Integrated analysis of periarticular tissues demonstrate concordance between the Multidimensional scaling (MDS) plot (Fig. 1a) and the Pearson correlation heatmap (Fig. 1b); with strong clustering of muscle samples, fat, capsule and synovium shown with bone samples scattered throughout, with total number of differentially expressed genes (DEG) up- or downregulated according to tissue type (Fig. 1c). Spearman correlation heatmap and colour histogram reveals strong clustering of samples by tissue type; Muscle, Fat, Capsule, Synovium, and Bone (Fig. 1d).

### Integrated analysis of periarticular tissue sample variation and clustering: MDS plot, correlation heatmaps, and differentially expressed genes

See Fig. 1

### Genes and gene pathways associated with increased QuickDASH score (worsening disability) according to tissue type

Genes downregulated in bone (Supplementary Table 3) are involved in GTPase activity and intracellular protein transport (*TBC1D3D*), immune responses (*IGLV8-61, NPIPB15*) and chondrocyte differentiation and survival (*SFRP2*). The most downregulated genes in capsule were *TBC1D3D, IGLV 3–10*, and the encoded protein part of MAC (*C6*), which is involved in cytolysis. Upregulated genes in capsule were *CXCL5*, *CXCL14*, *FCAR* and *SIK1* (Supplementary Table 3), which encode proteins involved in immune and inflammatory responses. Downregulation of immunoglobulin genes and upregulation of *SIK1* was observed in synovium, (Supplementary Fig. 2). Downregulation of *TBC1D3D* was observed in bone, capsule and fat, while, while *NPIPB15* was downregulated in bone and synovium (Supplementary Fig. 3).

**Fig. 2:**
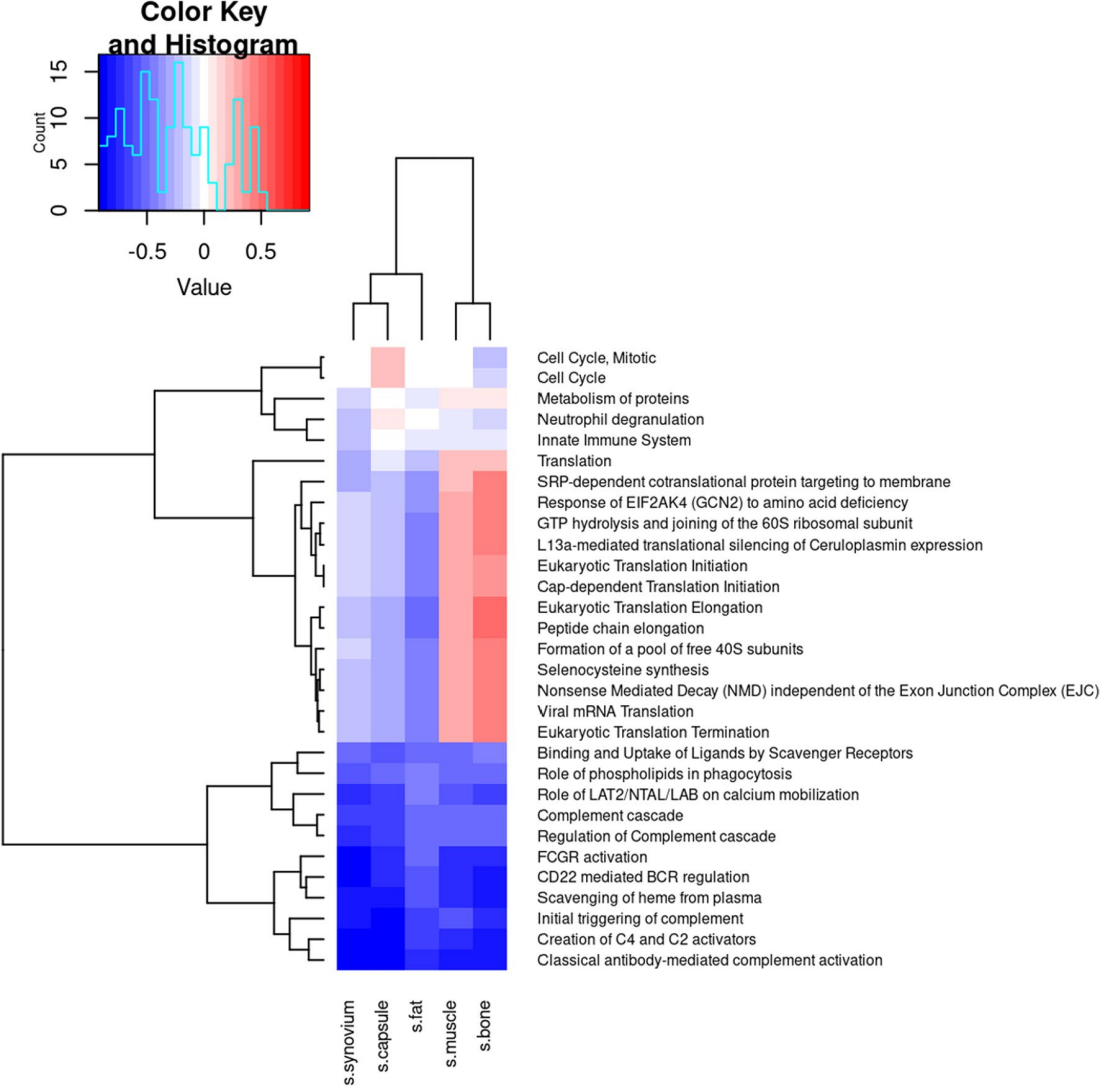
Heatmap of top gene pathways correlated with QuickDASH score common to all tissues, p.adjusted MANOVA < 0.000 for all pathways listed. Enrichment score is demonstrated on the colour key histogram. Blue indicates downregulation, and red upregulation of pathways listed

Downregulation of gene pathways common to all tissue types were dominated by complement associated pathways, others significant pathways are shown in Fig. 2.

**Fig. 3:**
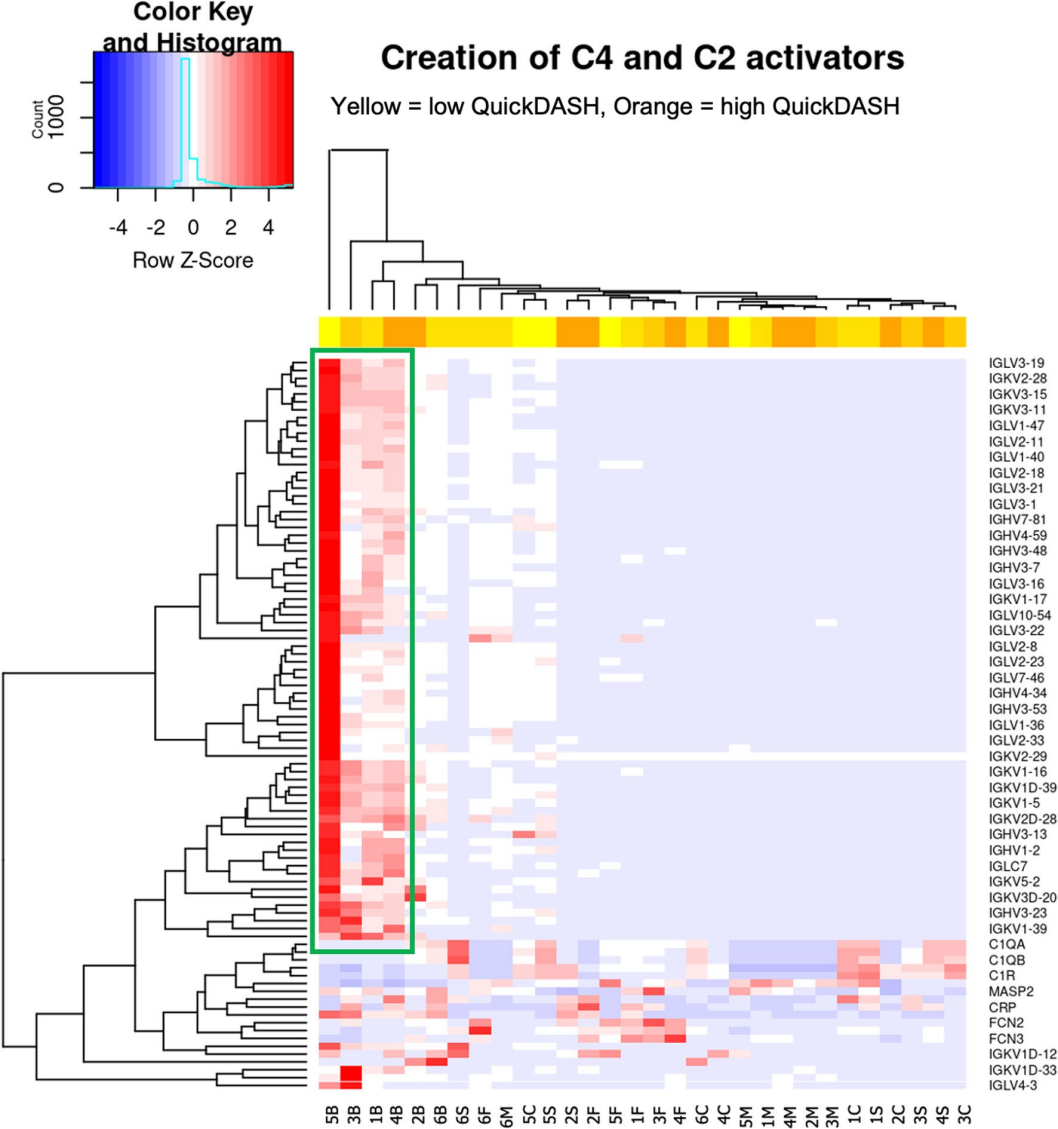
Hierarchical unsupervised (not ordered prior to analysis. Genes and samples are positioned based on their observed similarity) clustering gene expression heatmap and colour histogram demonstrating significant immunoglobulin expression (Y axis) in bone samples (X axis, 1B–6B) which reveal tight clustering and positive z score as encapsulated by the green box

Further pathway analysis using a gene expression heatmap demonstrated the expression of immunoglobulins in bone may explain the creation of *C4* and *C2* activators (Fig. 3, and supplementary Table 4).

### Genes and biological enrichment processes associated with OA compared with instability

With respect to the top differentially expressed genes, downregulation of mitochondrial associated genes dominated when comparing osteoarthritis to instability (Supplementary Fig. 3).

Comparing capsular biopsies, OA was characterised by downregulation of various signalling pathways, including those centred around the *ROR*α *(s. dist -0.696, p.adjustANOVA 0.000), RAC1 (s.dist -0.641, p.adjustANOVA 0.001), NOTCH2 (s.dist -0.673, p.adjustANOVA 0.001), CREB1 (s.dist -0.639, p.adjustANOVA 0.001)*, and *PI3K* genes (*s.dist -0.617, p.adjustANOVA 0.000).* Upregulated pathways in OA included translation, amino acid recycling, nonsense mediated decay, degradation of mitotic proteins and *P53* activators (p < 0.000, s.dist > 0.61) (Supplementary Fig. 4).

## Discussion

### Notable and novel genes associated with increasing (worsening) QuickDASH score in OA across tissue types

#### Synovium and capsule

Our study found NR4A3 upregulation, aligning with its known role in synovial hyperplasia, a key factor in OA pathogenesis. [20] Although *NR4A2* gene and protein expression contributes to synovial hyperplasia, *NR4A3* gene expression is equivalent in osteoarthritic and normal knees. [21] NR4A3 upregulation in our study might be a shoulder OA-specific biomarker. SIK1, normally active in cartilage, was downregulated in knee OA but upregulated in shoulder OA, indicating inflammation (Supplementary Figs. 10, 13). Because *SIK* inhibition is osteoclastogenic [22], future studies might explore potential relationships with the ‘bone former’ OA phenotype, and upregulation with subchondral cyst formation, or ‘bone resorber’ and OA. Additionally, CXCL5 (supplementary Fig. 13), linked to joint inflammation [23], and *CXCL2* in muscle (supplementary Fig. 11), and *CXCL8* in fat (supplementary Fig. 12) were proinflammatory genes associated with worsening shoulder function and pain.

#### Fat

Zinc dependant transcription signalling causes OA progression after biomechanical injury in vivo via inflammatory mediators [24] and downregulation of FEZF2 in our study is, a novel finding in shoulder OA (Supplementary Table 3, supplementary Fig. 12). TBC1D3, which delays wound healing by altering extracellular vesicle delivery [25], was also downregulated, suggesting its potential as a biomarker for disease progression (Supplementary Table 3, supplementary Fig. 12).

#### Subchondral bone

SFRP2, which regulates chondrocyte differentiation and survival, was downregulated, consistent with findings in murine OA models (Supplementary Fig. 14). [26] Downregulation may contribute to extracellular matrix breakdown [27], through increased MMP13 expression [28], and in our study confirms its relevance in human shoulder OA.

### Genes and biological processes associated with increasing (worsening) QuickDASH score across tissue types in shoulder OA

The common reactome pathways linked to worsening QuickDASH score show a downregulation of immune, inflammatory, and host defence mechanisms. Cell cycle and mitotic pathways were upregulated in the joint capsule of OA patients (see supplementary Figs. 4 and 6), indicating a complex interplay of inflammation, cell proliferation, and tissue remodelling in end-stage disease. A key pathway downregulated across multiple tissues (capsule, bone, muscle, fat, synovium, see supplementary Figs. 5, 7 and 9) was the scavenging of heme from plasma, previously linked to knee OA pathogenesis and inflammation [29], suggesting a common pathway in shoulder OA that requires further investigation.

In the synovium, pathways tied to chronic inflammation, immune dysregulation, and metabolic disturbances were prominently downregulated (Supplementary Fig. 9). Synovial inflammation, known to drive fibrosis and capsular contracture in OA [30, 31], aligns with upregulated cell cycle and mitotic pathways linked to worse QuickDASH scores, emphasizing the role of capsular tissue in disease progression.

Complement pathways were differentially expressed across all tissue types, suggesting local complement activation (Fig. 2 and supplementary Figs. 5–9). This aligns with Assirelli et al., who described complement activation fragments in cartilage and synovium occurring without parent molecules from the bloodstream. [7] Further post hoc analysis showed that immunoglobulins in bone may create C4 and C2 activators (Fig. 3, and supplementary Table 4); explicably, contamination with blood is thought less likely given similar findings in fat, a relatively avascular tissue. The creation of C4 and C2 activators primarily involves cleavage and activation of complement components, leading to the formation of the C3 convertase enzyme complex. This pathway was further analysed to understand complement activation within the synovial joint complex. We posit that the C3 convertase enzyme complex, essential for the complement cascade and inflammation, is responsible for the injurious sequelae of complement activation in periarticular tissues in shoulder OA; and through negative feedback inhibition downregulated C4 and C2 activators in end-stage disease.

### Significant differentially expressed genes in shoulder OA (capsular tissue) compared with instability

Targeting mitochondrial dysfunction has been proposed for the treatment of OA. [32] In knee OA, cartilage degeneration can result from diminished ATP production [33], increased oxidative stress [34, 35], and calcium dysregulation. [36] Downregulation of the following mitochondrial genes in association with worsening shoulder function in our OA cohort is novel: *MT-TE*, *MT-TY* and *Mt-ts1* (Supplementary Fig. 3). Of the top 20 expressed genes, *ATP5MD* was singularly upregulated in our study (Supplementary Fig. 3), further the tricarboxylic acid pathway is generally upregulated in OA, probably to increase ATP production for cellular repair, which is more pronounced in late disease. [37].

### Biological processes downregulated in OA (capsular tissue), compared with instability: pathways linked to ROR*α*, RAC1, NOTCH2, CREB1, and PI3K

RORα, RAC1, NOTCH2, and CREB1 are upregulated in knee OA. [38–40] Although we anticipated the activation of inflammatory pathways, signalling and surveillance pathways were most significantly differentially expressed in OA compared to instability (see supplementary Fig. 3). RORα has some inflammatory potential, as IL-6/STAT3 pathway downregulation occurs upon RORα blockade in mice. [39] NOTCH2 sensitizes chondrocytes to TNFα’s inflammatory action [41], and sustained NOTCH1 activation promotes an OA phenotype, while transient activation is chondroprotective, [42] suggesting a potential target for DMOADs.

The downregulation of the nonsense-mediated decay (NMD) pathway in OA may be driven by chronic inflammation, oxidative stress, ER stress, and aging, reducing the degradation of faulty mRNAs and contributing to harmful truncated proteins. Aberrant RAC1 activity is linked to chondrocyte hypertrophy and mineralization, precursors to OA, with OCRL-1 injection showing protective effects in murine models. [43] Activated RAC1 is significantly upregulated in OA knees, enhancing chondrocyte hypertrophy and matrix degradation in vitro. [44] Whether inhibition of Rac1 activity in articular chondrocytes in humans has the potential to delay OA development in humans is an area for future study.

PI3K/AKT/mTOR signalling, which promotes chondrocyte proliferation and reduces apoptosis [45], is inhibited in knee OA. [46] We observed downregulation of PI3K and KAT6A in capsular tissue of osteoarthritic shoulders, consistent with disease mechanisms described in other joints. [45, 47, 48].

### Limitations

Despite varying baseline QuickDASH scores (38.6—86.4), our transcriptomic inferences about worsening shoulder disability may not apply across the disease spectrum. Obtaining tissue samples at different disease stages could help identify unique transcriptomic patterns over time.

The tight clustering in the MDS plot (Fig. 1a) and sufficient read counts (> 15 million for all but four samples) suggest the low yield of differentially expressed genes in the OA group (N = 6) is likely due to the small sample size rather than similar pathway expression. Both parametric and non-parametric analyses showed similar results, indicating a negligible batch effect; non-parametric results were reported due to the small sample size.

Shoulders with instability exhibit an anabolic rather than inflammatory or catabolic phenotype [9], allowing gene comparisons after a quiescent period post-trauma. Despite age and sex differences between groups, prior work showed no significant relationship between gene expression and these factors, supporting the use of instability as a control. [49] While recurrent shoulder instability is a known OA risk factor, the contribution of initial trauma, recurrent episodes, or other mechanisms remains unclear. Excluding patients with a history of instability in the OA group assumes that genetic changes from instability are unrelated to OA.

Metabolic syndrome-associated OA is recognized, but its mechanisms are unproven. [50] Our study found clear between-group differences in metabolic syndrome risk factors (BMI, HbA1c, hypercholesterolemia, hypertension) when comparing OA and instability. Larger studies are needed to explore transcriptomic changes in metabolic syndrome patients with and without OA before interpreting gene and pathway differences related to inflammation, protein synthesis, and mitochondrial genes as integral to OA rather than metabolic syndrome.

## Supplementary Information

Supplementary material 1: Supplementary figure 1. Quality Control analysis. There were 4 samples with fewer than 15M reads: 2S, 6B, 2F and 6C

Supplementary material 2: Supplementary figure 2. Figure Volcano plot with log2FoldChange in the horizontal coordinate and -log_10_(P-value) in the vertical coordinate, of significantly differentially DEGs in shoulder OA compared with instability. Red nodes indicate upregulated DEGs with FDR of 0.05.

Supplementary material 3: Supplementary figure 3. Top 20 differentially expressed genes in capsular tissue biopsies between OA (case) and instability (control). Mitochondrial related genes exhibited the highest fold change of all significantly downregulated genes.

Supplementary material 4: Supplementary figure 4. Biological enrichment processes in OA compared with instability.

Supplementary material 5: Supplementary figure 5. Top ranked biological enrichment processes in bone associated with QuickDASH score. Pathways were prioritised based on the magnitude of enrichment score after removing sets with FDR>0.05.

Supplementary material 6: Supplementary figure 6. Top ranked biological enrichment processes in capsule associated with QuickDASH score. Pathways were prioritised based on the magnitude of enrichment score after removing sets with FDR>0.05.

Supplementary material 7: Supplementary figure 7. Top ranked biological enrichment processes in muscle associated with QuickDASH score. Pathways were prioritised based on the magnitude of enrichment score after removing sets with FDR>0.05.

Supplementary material 8: Supplementary figure 8. Top ranked biological enrichment processes in fat associated with QuickDASH score. Pathways were prioritised based on the magnitude of enrichment score after removing sets with FDR>0.05.x.

Supplementary material 9: Supplementary figure 9. Top ranked biological enrichment processes in synovium associated with QuickDASH score. Pathways were prioritised based on the magnitude of enrichment score after removing sets with FDR>0.05.

Supplementary material 10: Supplementary figure 10. Hierarchical clustering gene expression heatmap and colour histogram demonstrating top differentially expressed genes in synovium associated with worsening (increased) QuickDASH score.

Supplementary material 11: Supplementary figure 11. Hierarchical clustering gene expression heatmap and colour histogram demonstrating top differentially expressed genes in muscle associated with worsening (increased) QuickDASH score.

Supplementary material 12: Supplementary figure 12. Hierarchical clustering gene expression heatmap and colour histogram demonstrating top differentially expressed genes in fat associated with worsening (increased) QuickDASH score.

Supplementary material 13: Supplementary figure 13. Hierarchical clustering gene expression heatmap and colour histogram demonstrating top differentially expressed genes in capsule associated with worsening (increased) QuickDASH score.

Supplementary material 14: Supplementary figure 14. Hierarchical clustering gene expression heatmap and colour histogram demonstrating top differentially expressed genes in bone associated with worsening (increased) QuickDASH score.

Supplementary material 15: Supplementary table 1. Pre- and post-operative patient reported QuickDASH score between groups across various time points. Student’s t-tests were performed for continuous data.

Supplementary material 16: Supplementary table 2. Baseline preoperative characteristics in the osteoarthritis and instability groups. All data rounded to 3 decimal places. Student’s t-tests were performed for continuous data.

Supplementary material 17: Supplementary table 3. Top genes correlating with QuickDASH according to periarticular tissue with minimal significant fold change. All data rounded to 3 decimal places.

Supplementary material 18: Supplementary table 4. Top 20 genes, creation of C4 and C2 activators. The Gene Rank is based on the rank of the DESeq2 test statistic value, centred around zero which determines no change in expression.

## Data Availability

The data discussed in this publication have been deposited in NCBI’s Gene Expression Omnibus (Edgar R, Domrachev M, Lash AE.Gene Expression Omnibus: NCBI gene expression and hybridization array data repository Nucleic Acids Res. 2002 Jan 1;30(1):207-10) and are accessible through GEO Series accession number GSE281476 (https://www.ncbi.nlm.nih.gov/geo/query/acc.cgi?acc = GSE281476).
