# Supplementary figures and images for: ‘QuickDASH’ to find unique genes and biological processes associated with shoulder osteoarthritis: a prospective case–control study
Source: BMC Res Notes. 2024 Dec 19;17:361. doi: 10.1186/s13104-024-07035-9 (PMC11657115; doi:10.1186/s13104-024-07035-9)

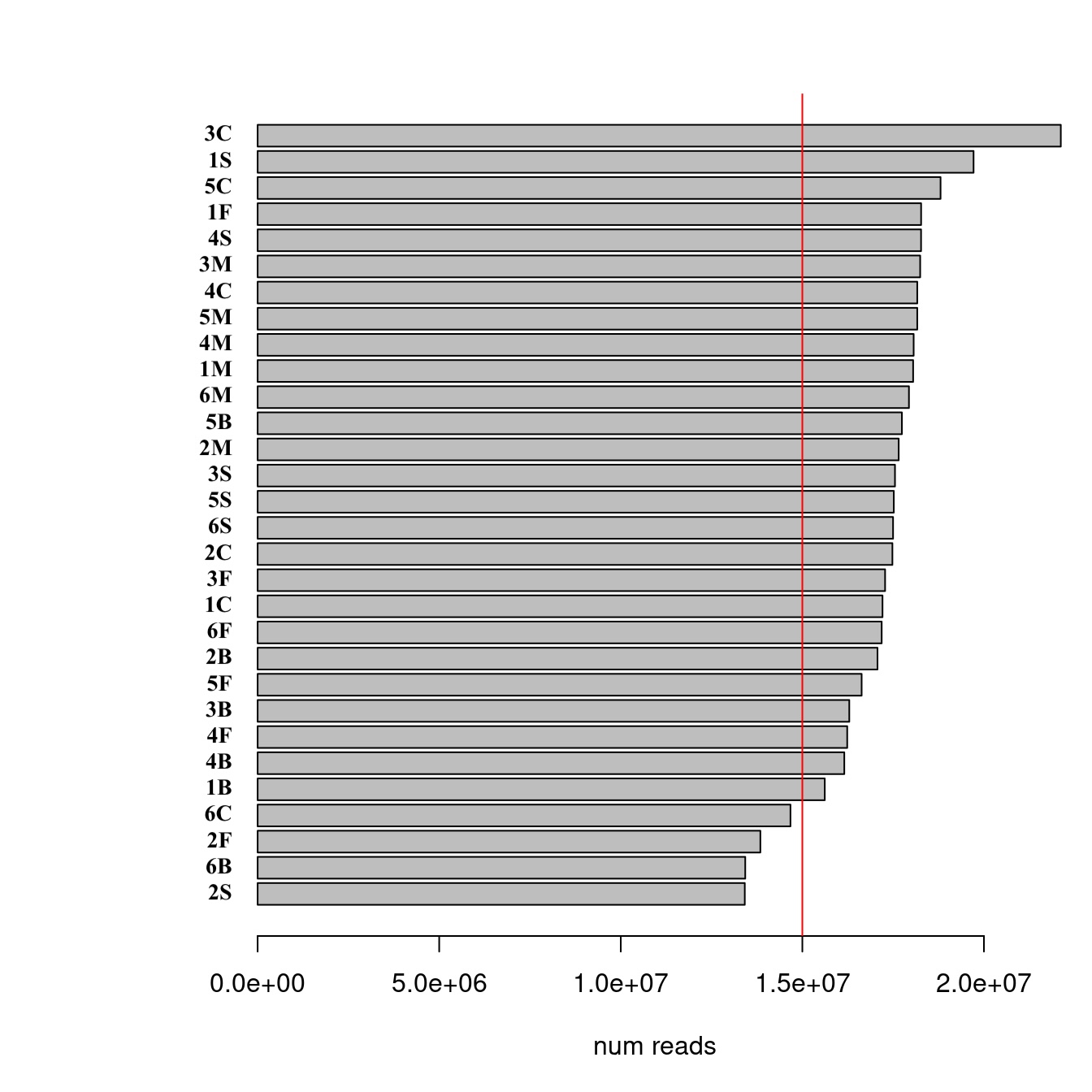

Supplement: Supplementary file 1 — Supplementary material 1: Supplementary figure 1. Quality Control analysis. There were 4 samples with fewer than 15M reads: 2S, 6B, 2F and 6C [file 13104_2024_7035_MOESM1_ESM.jpg]

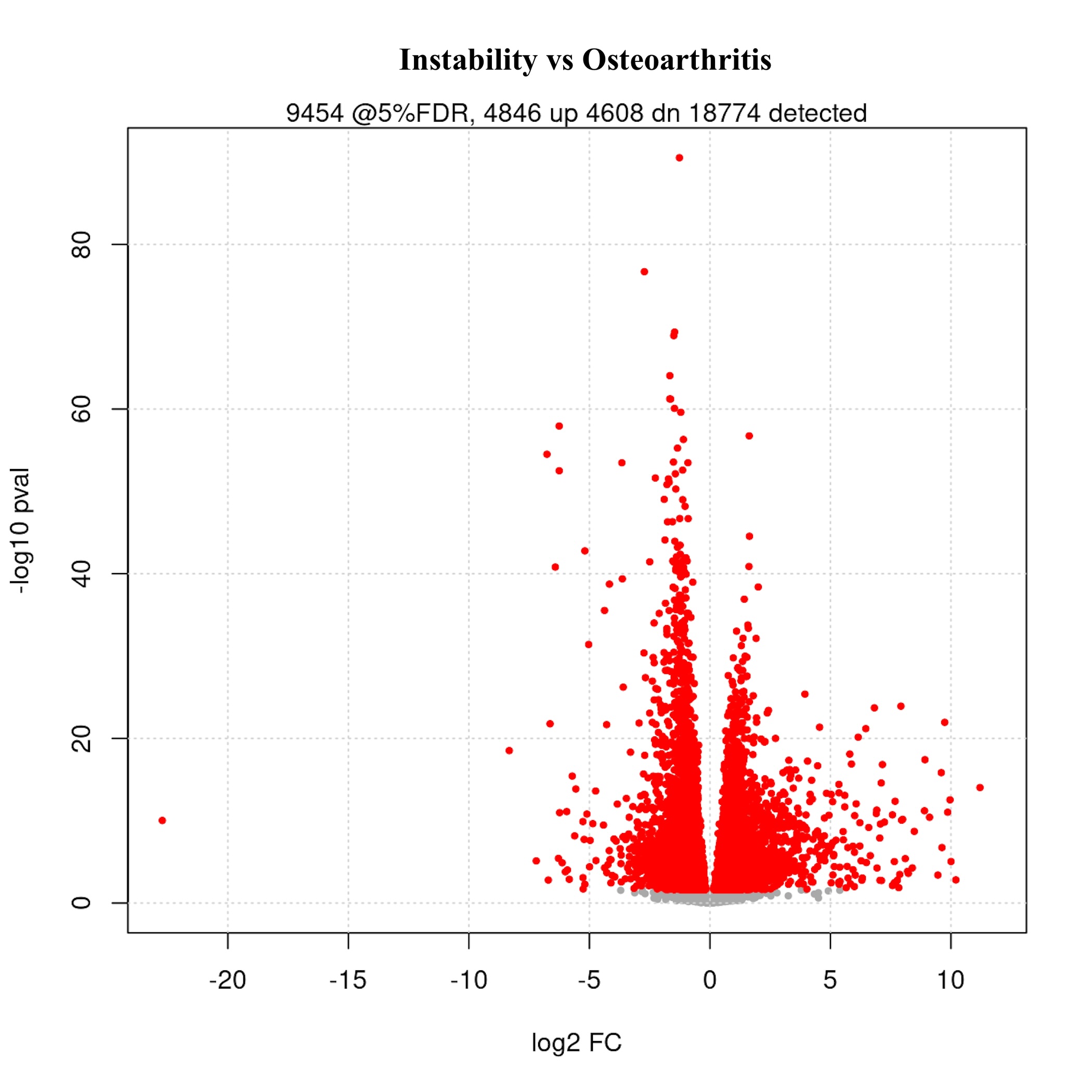

Supplement: Supplementary file 2 — Supplementary material 2: Supplementary figure 2. Figure Volcano plot with log2FoldChange in the horizontal coordinate and -log10(P-value) in the vertical coordinate, of significantly differentially DEGs in shoulder OA compared with instability. Red nodes indicate upregulated DEGs with FDR of 0.05. [file 13104_2024_7035_MOESM2_ESM.jpg]

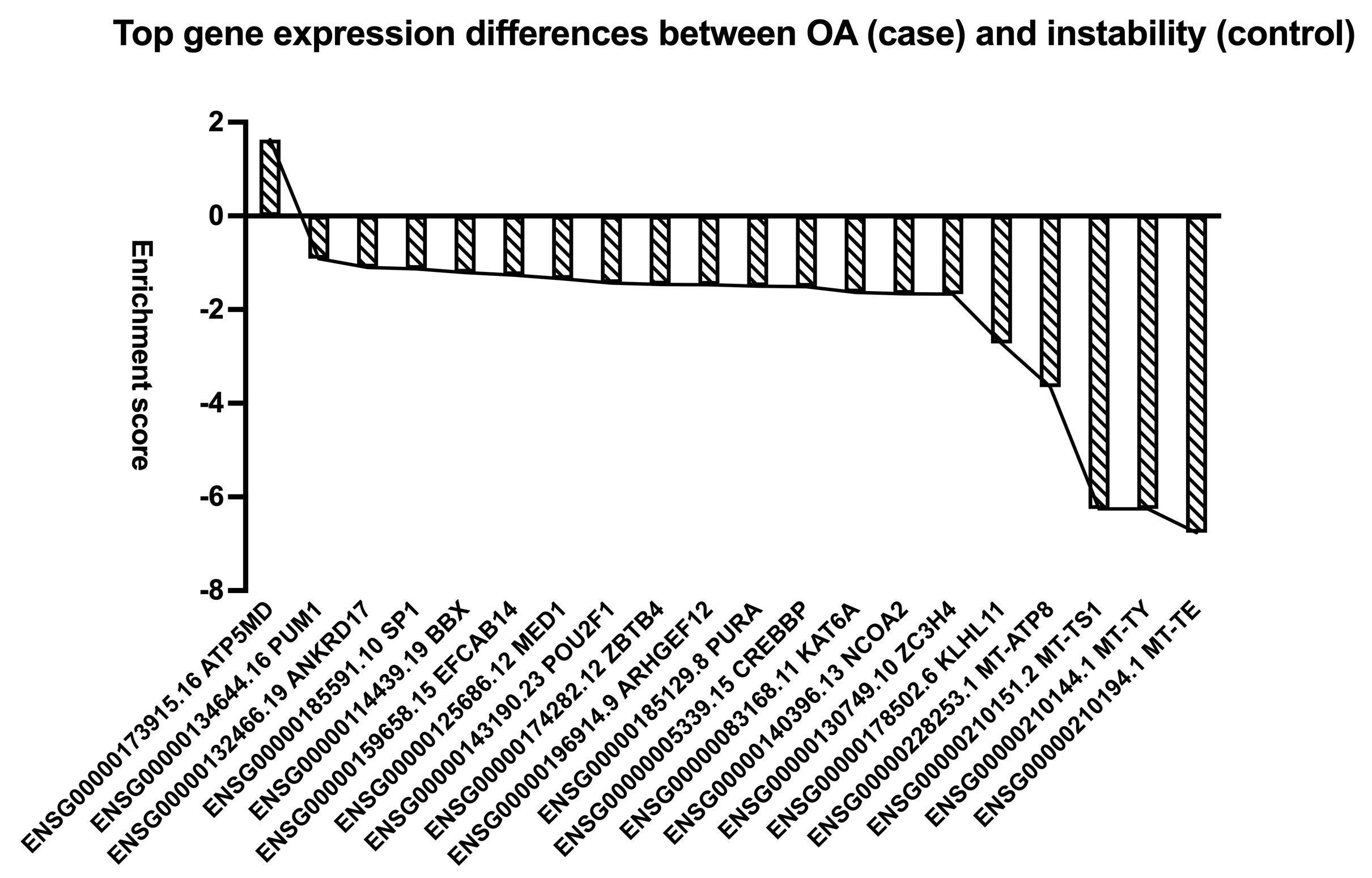

Supplement: Supplementary file 3 — Supplementary material 3: Supplementary figure 3. Top 20 differentially expressed genes in capsular tissue biopsies between OA (case) and instability (control). Mitochondrial related genes exhibited the highest fold change of all significantly downregulated genes. [file 13104_2024_7035_MOESM3_ESM.jpg]

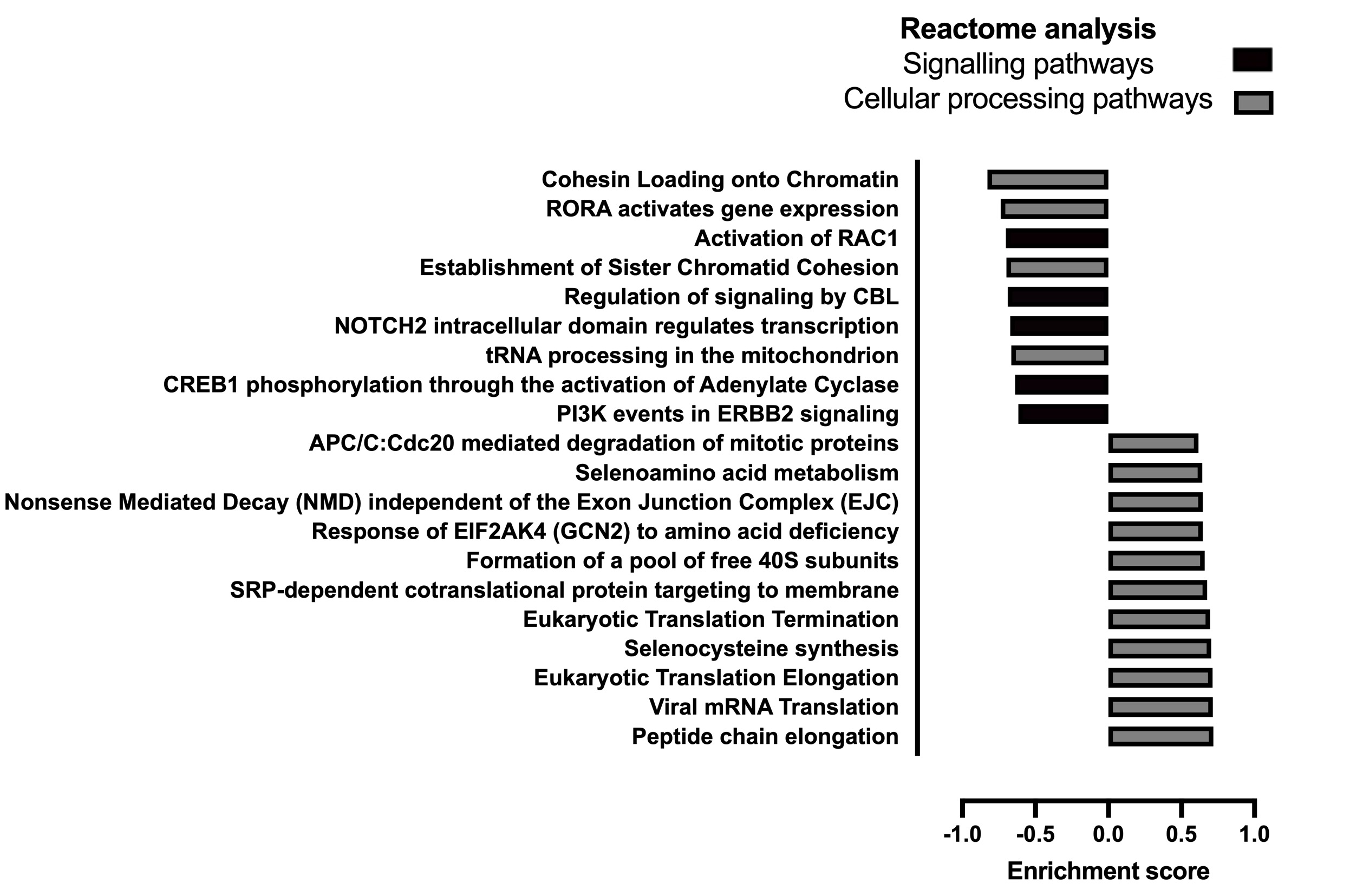

Supplement: Supplementary file 4 — Supplementary material 4: Supplementary figure 4. Biological enrichment processes in OA compared with instability. [file 13104_2024_7035_MOESM4_ESM.jpg]

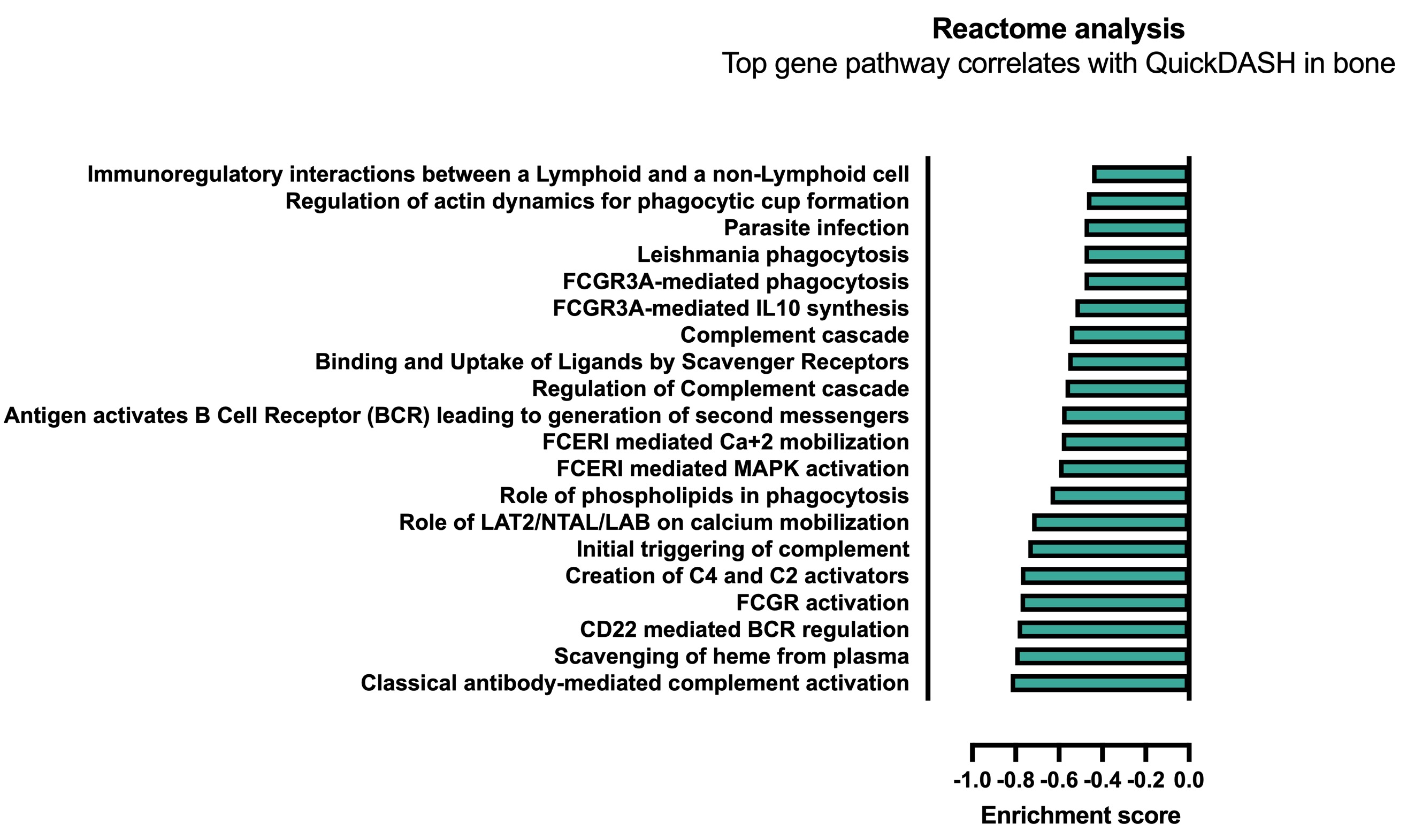

Supplement: Supplementary file 5 — Supplementary material 5: Supplementary figure 5. Top ranked biological enrichment processes in bone associated with QuickDASH score. Pathways were prioritised based on the magnitude of enrichment score after removing sets with FDR>0.05. [file 13104_2024_7035_MOESM5_ESM.jpg]

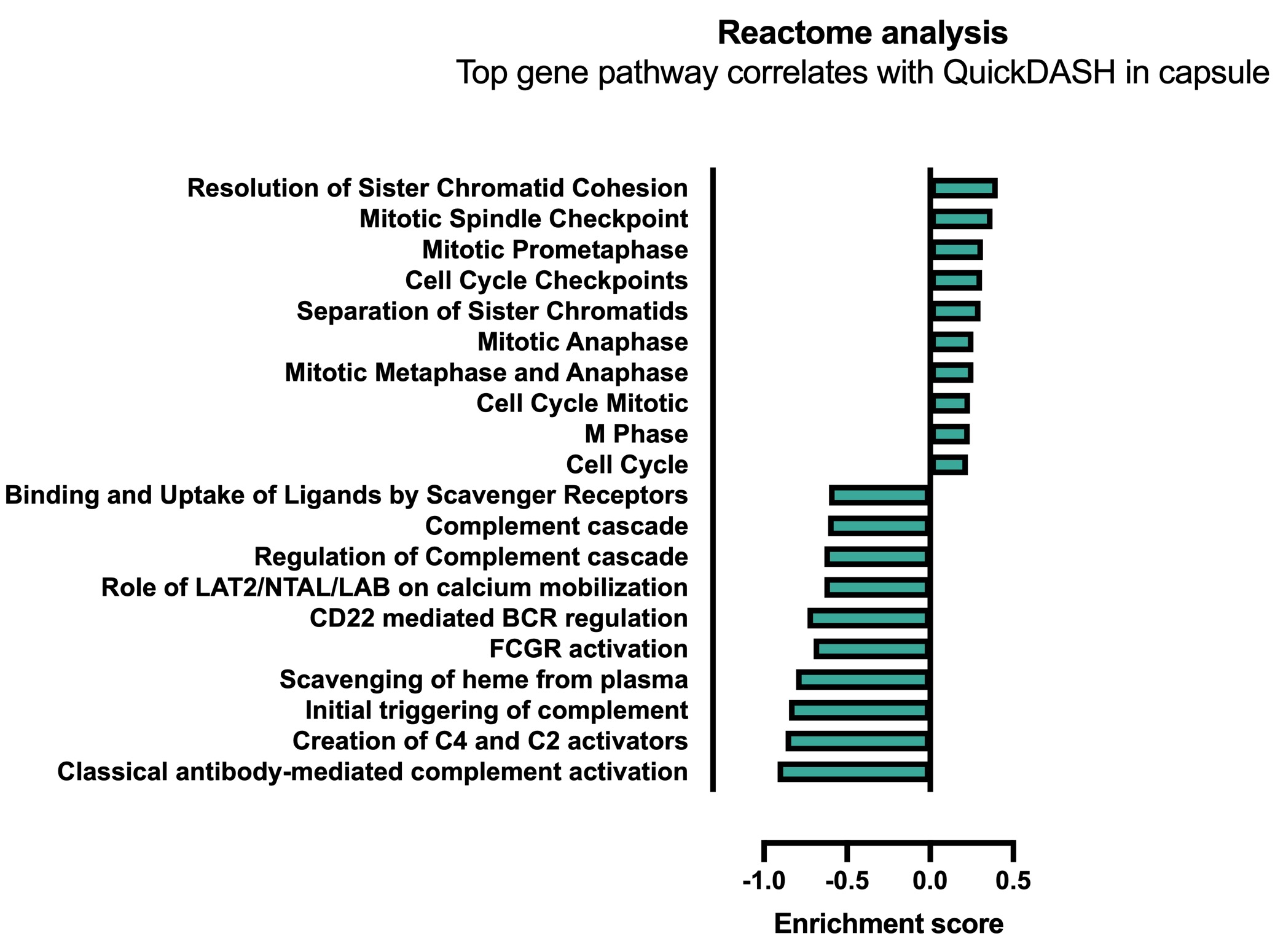

Supplement: Supplementary file 6 — Supplementary material 6: Supplementary figure 6. Top ranked biological enrichment processes in capsule associated with QuickDASH score. Pathways were prioritised based on the magnitude of enrichment score after removing sets with FDR>0.05. [file 13104_2024_7035_MOESM6_ESM.jpg]

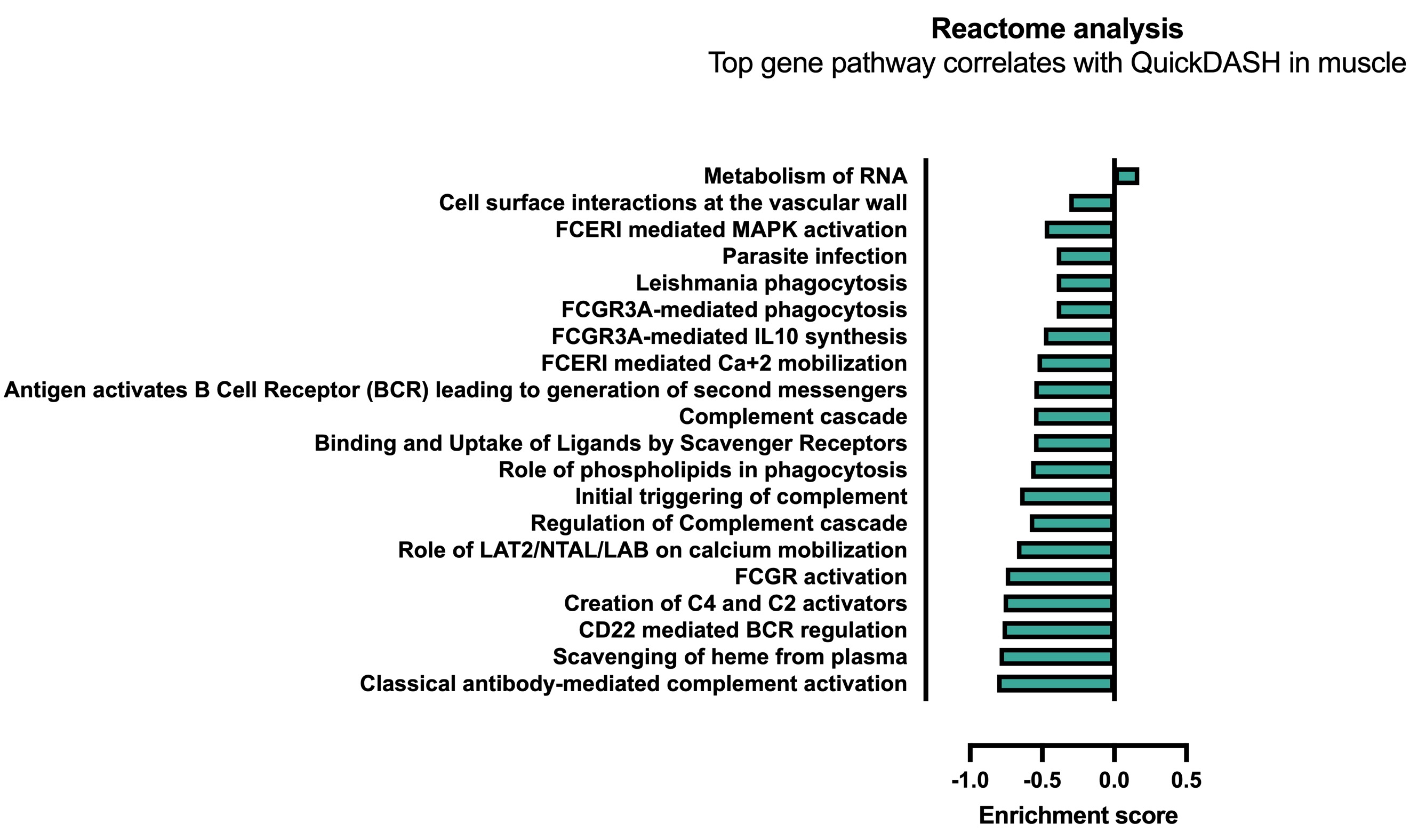

Supplement: Supplementary file 7 — Supplementary material 7: Supplementary figure 7. Top ranked biological enrichment processes in muscle associated with QuickDASH score. Pathways were prioritised based on the magnitude of enrichment score after removing sets with FDR>0.05. [file 13104_2024_7035_MOESM7_ESM.jpg]

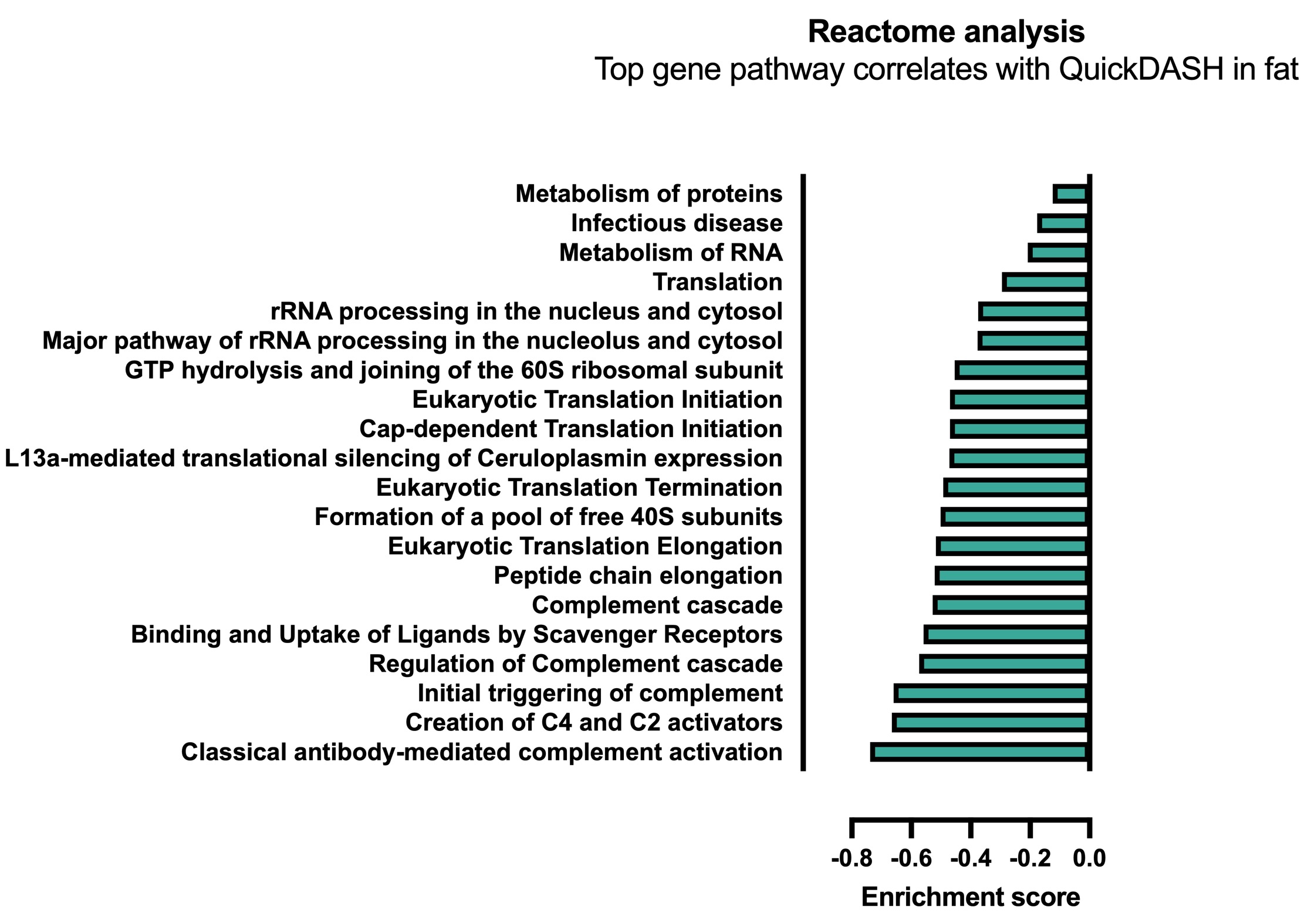

Supplement: Supplementary file 8 — Supplementary material 8: Supplementary figure 8. Top ranked biological enrichment processes in fat associated with QuickDASH score. Pathways were prioritised based on the magnitude of enrichment score after removing sets with FDR>0.05.x. [file 13104_2024_7035_MOESM8_ESM.jpg]

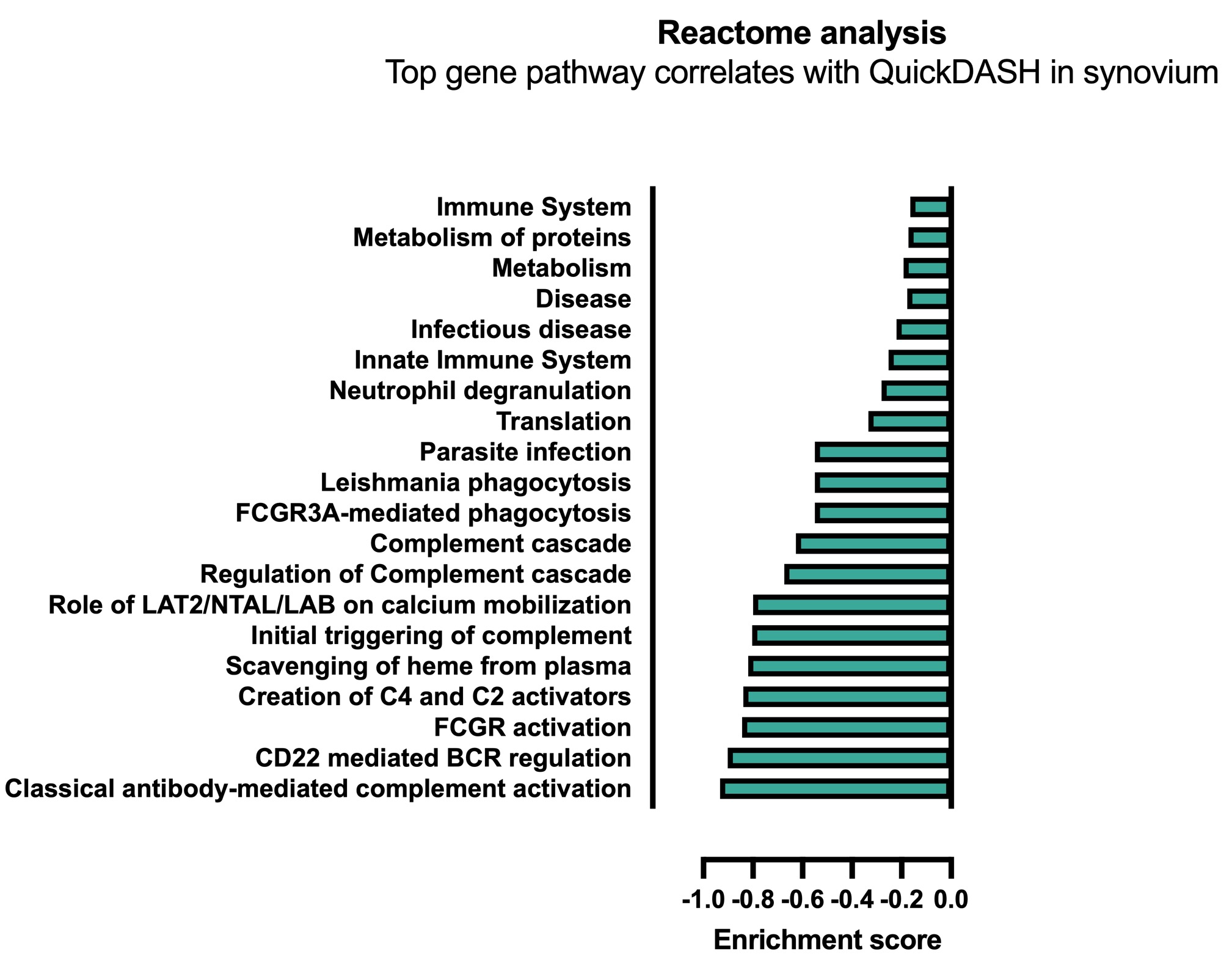

Supplement: Supplementary file 9 — Supplementary material 9: Supplementary figure 9. Top ranked biological enrichment processes in synovium associated with QuickDASH score. Pathways were prioritised based on the magnitude of enrichment score after removing sets with FDR>0.05. [file 13104_2024_7035_MOESM9_ESM.jpg]

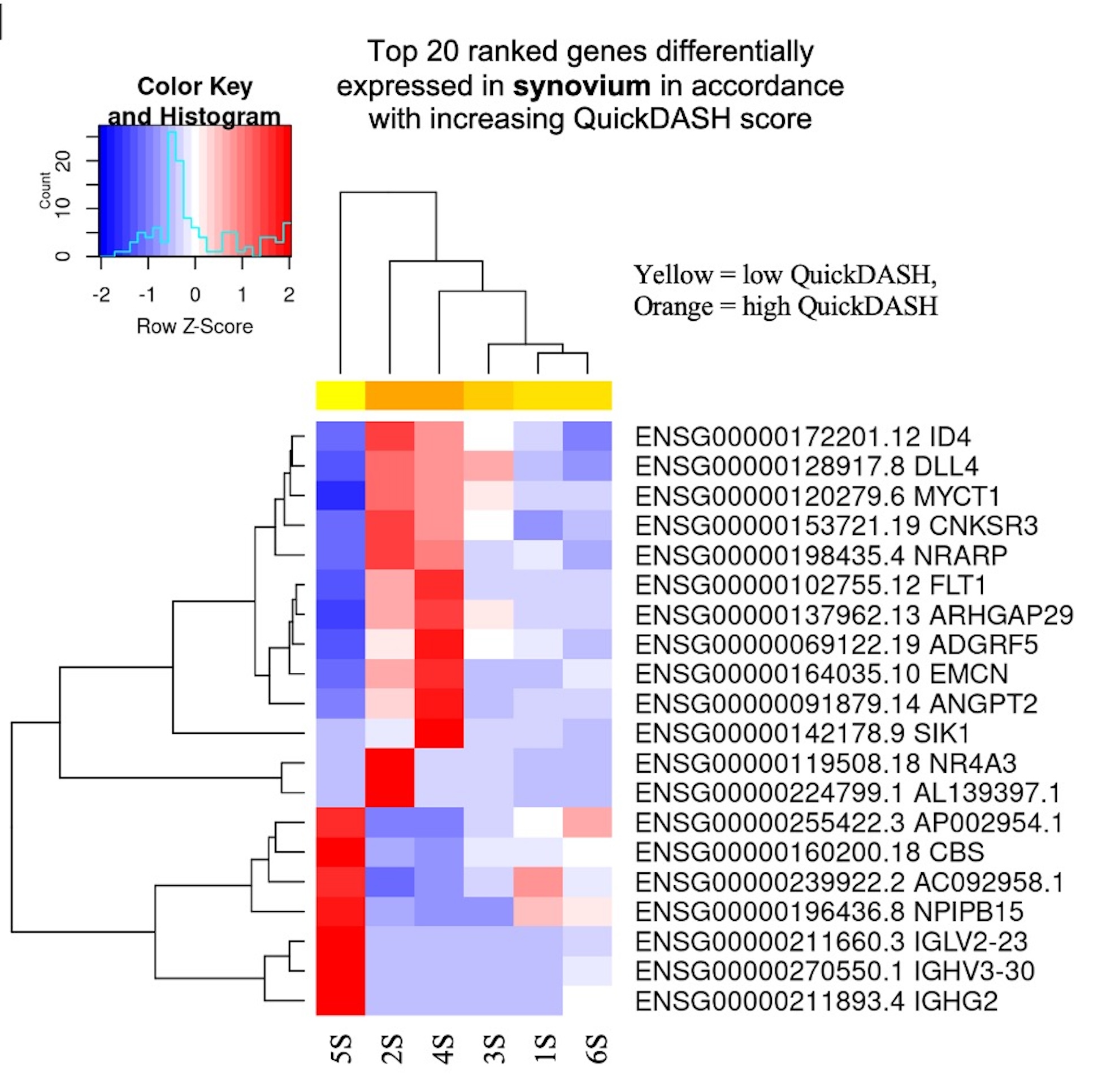

Supplement: Supplementary file 10 — Supplementary material 10: Supplementary figure 10. Hierarchical clustering gene expression heatmap and colour histogram demonstrating top differentially expressed genes in synovium associated with worsening (increased) QuickDASH score. [file 13104_2024_7035_MOESM10_ESM.jpg]

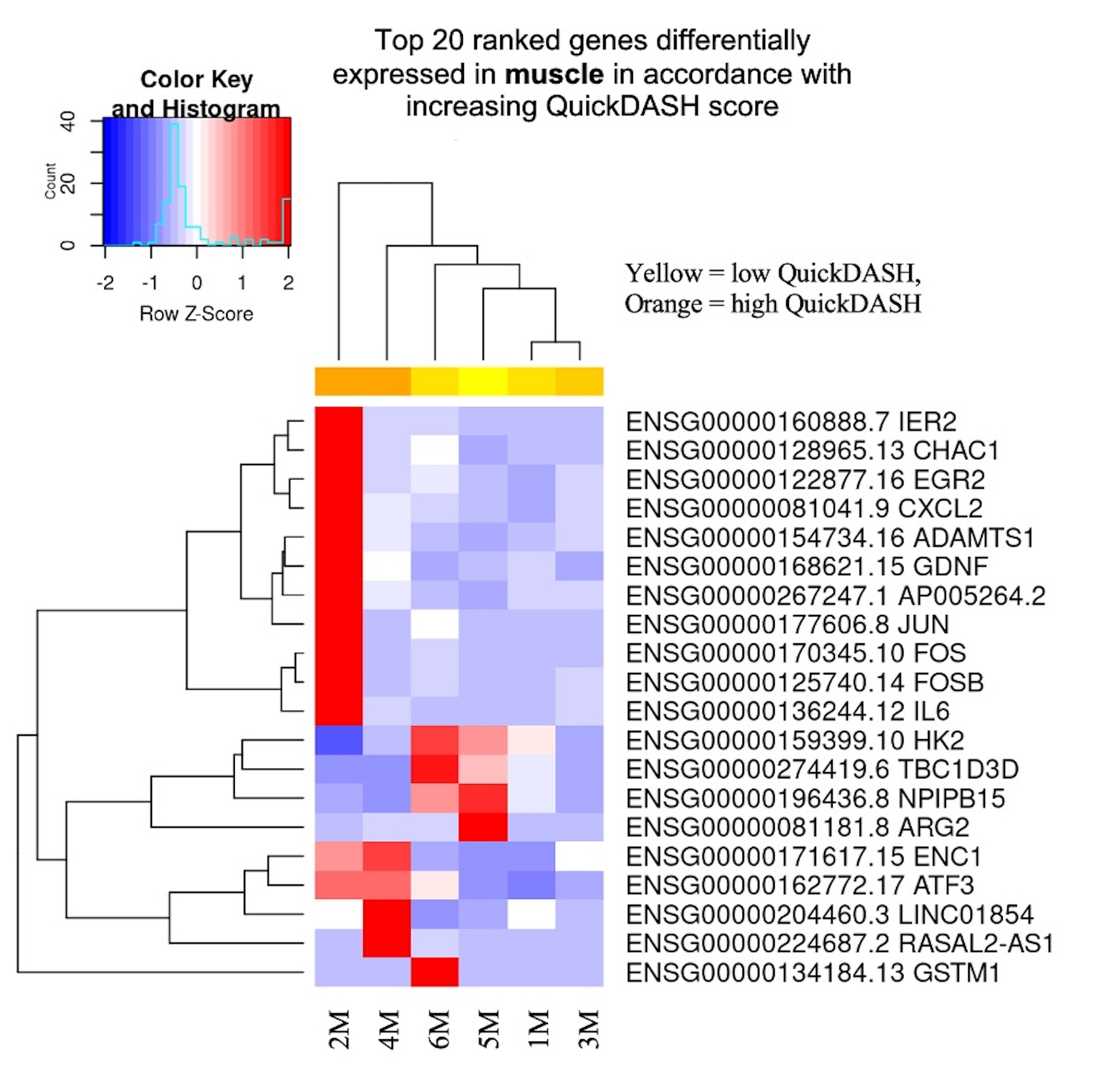

Supplement: Supplementary file 11 — Supplementary material 11: Supplementary figure 11. Hierarchical clustering gene expression heatmap and colour histogram demonstrating top differentially expressed genes in muscle associated with worsening (increased) QuickDASH score. [file 13104_2024_7035_MOESM11_ESM.jpg]

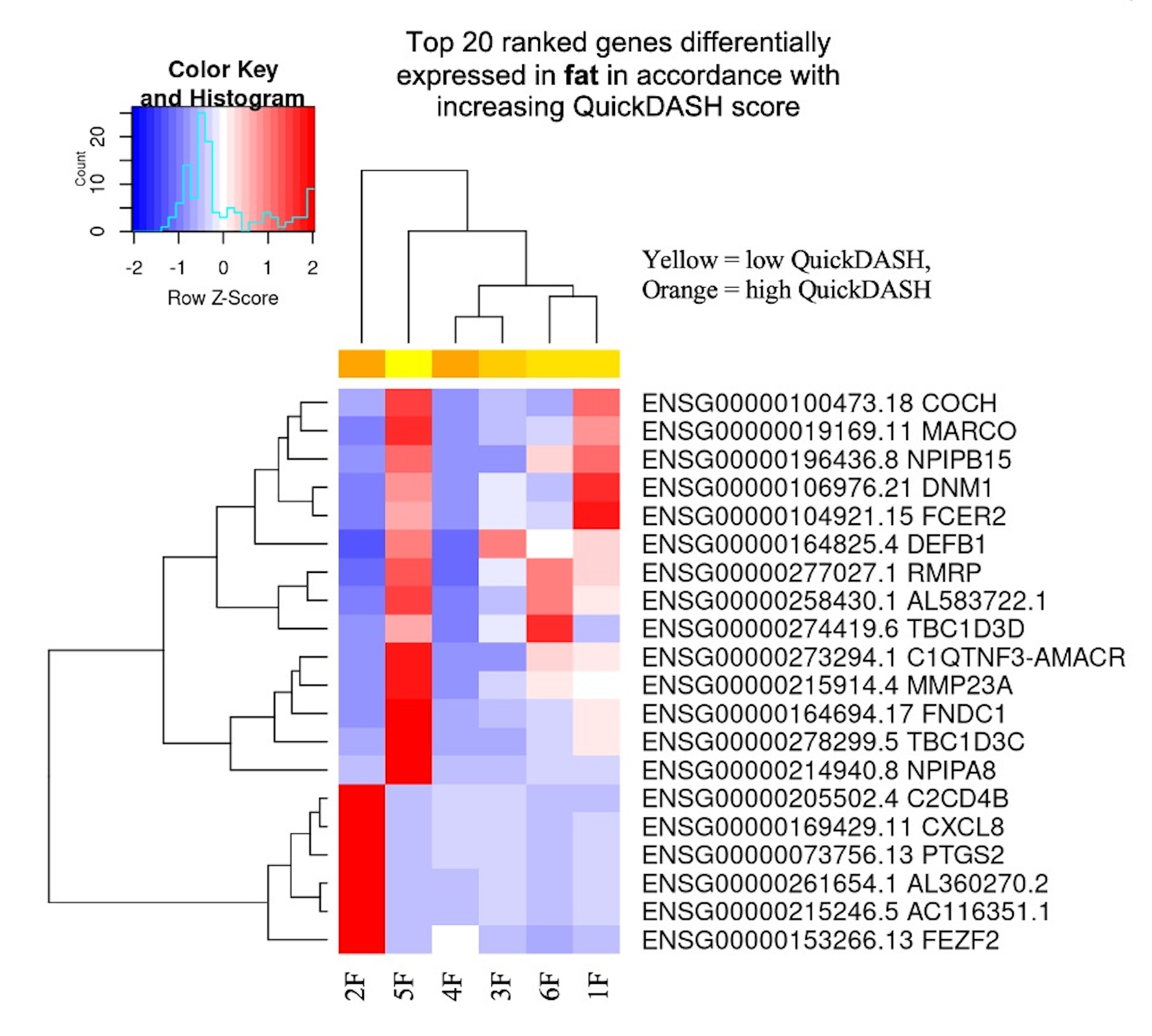

Supplement: Supplementary file 12 — Supplementary material 12: Supplementary figure 12. Hierarchical clustering gene expression heatmap and colour histogram demonstrating top differentially expressed genes in fat associated with worsening (increased) QuickDASH score. [file 13104_2024_7035_MOESM12_ESM.jpg]

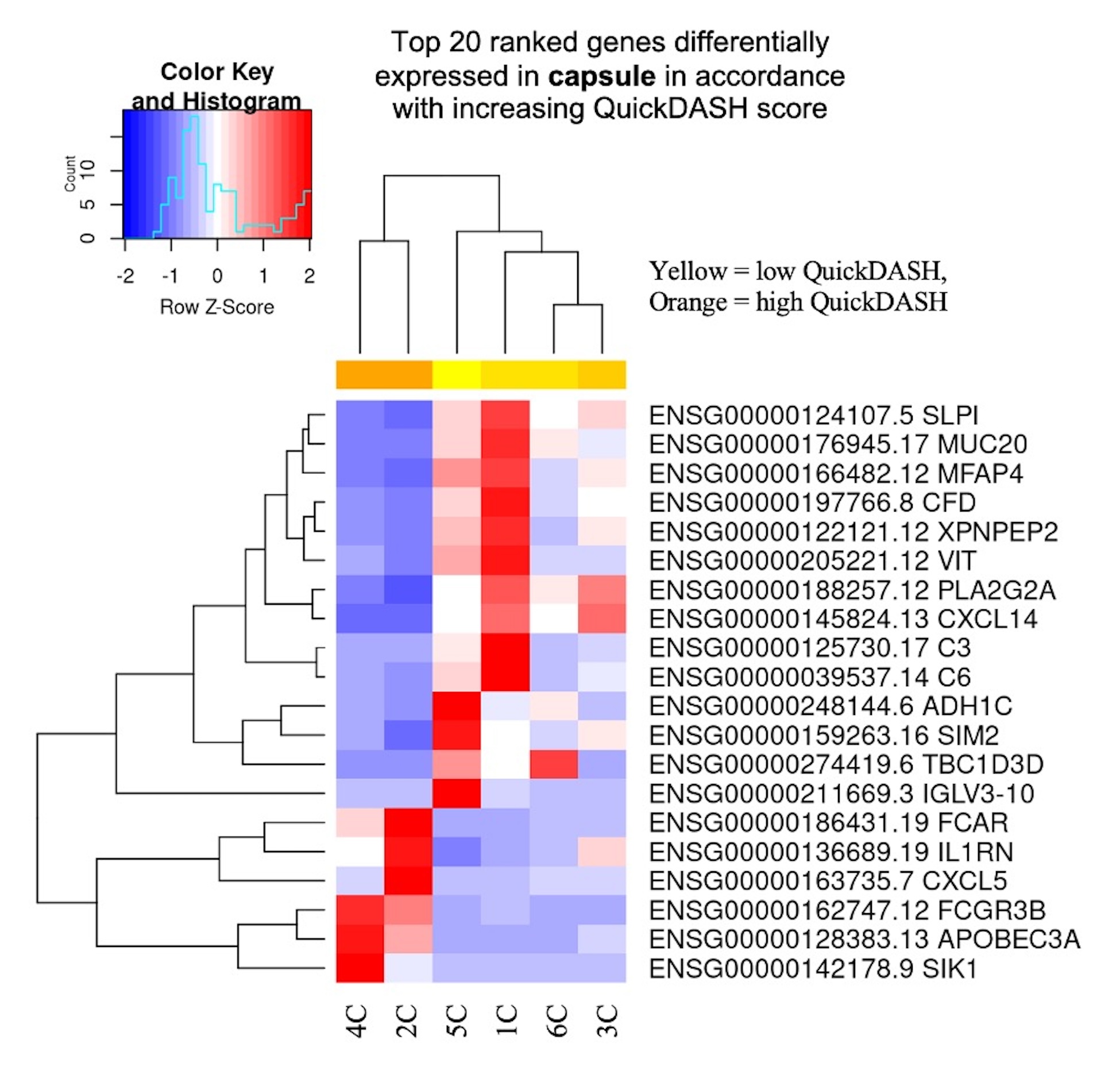

Supplement: Supplementary file 13 — Supplementary material 13: Supplementary figure 13. Hierarchical clustering gene expression heatmap and colour histogram demonstrating top differentially expressed genes in capsule associated with worsening (increased) QuickDASH score. [file 13104_2024_7035_MOESM13_ESM.jpg]

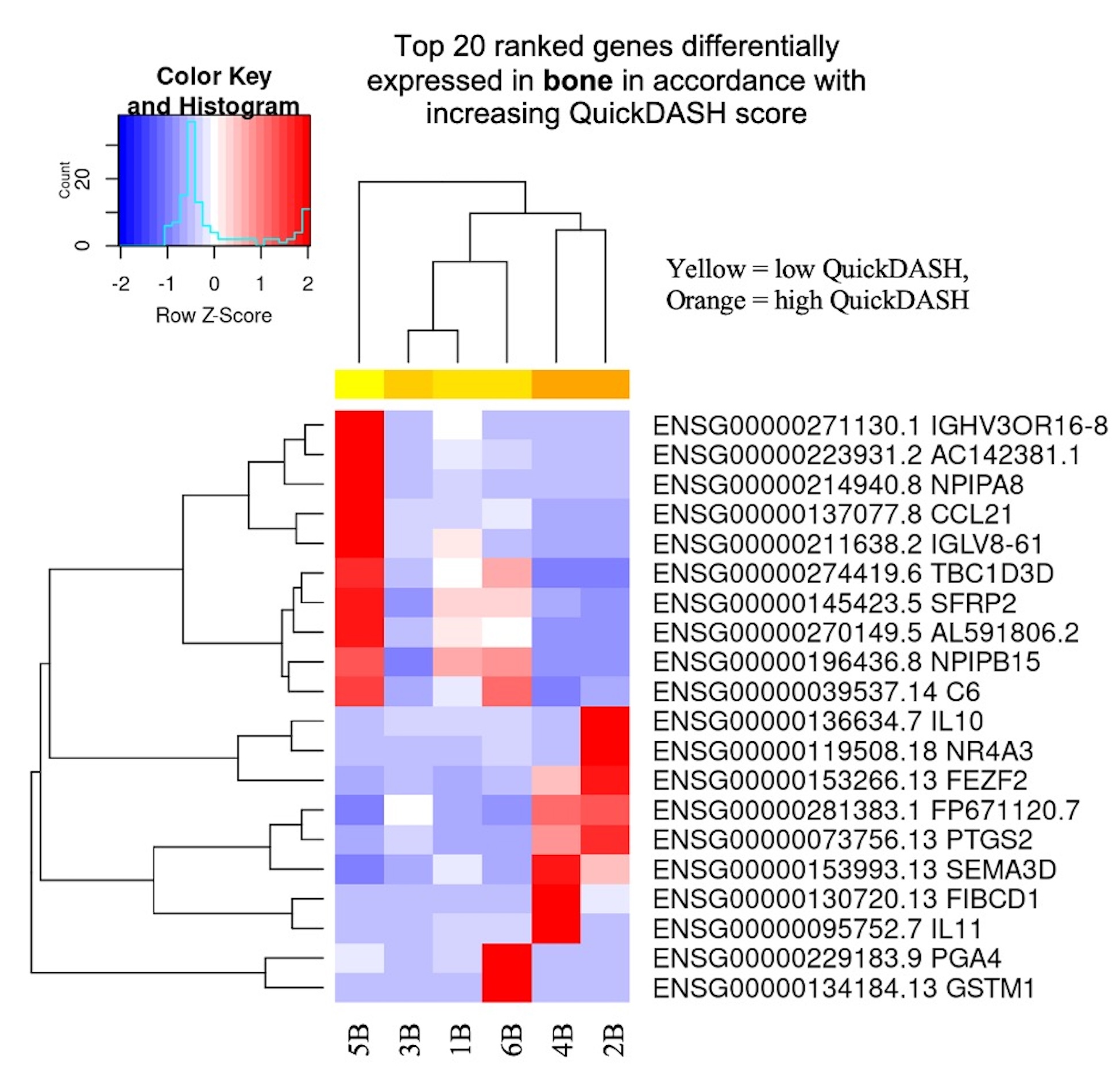

Supplement: Supplementary file 14 — Supplementary material 14: Supplementary figure 14. Hierarchical clustering gene expression heatmap and colour histogram demonstrating top differentially expressed genes in bone associated with worsening (increased) QuickDASH score. [file 13104_2024_7035_MOESM14_ESM.jpg]
