# Supplementary material for: ‘QuickDASH’ to find unique genes and biological processes associated with shoulder osteoarthritis: a prospective case–control study
Source: BMC Res Notes. 2024 Dec 19;17:361. doi: 10.1186/s13104-024-07035-9 (PMC11657115; doi:10.1186/s13104-024-07035-9)
Supplement: Supplementary file 15 — Supplementary material 15: Supplementary table 1. Pre- and post-operative patient reported QuickDASH score between groups across various time points. Student’s t-tests were performed for continuous data. [file 13104_2024_7035_MOESM15_ESM.docx]

| Patient reported outcome measure | Osteoarthritis  mean (range) | Instability  mean (range) | p value |
| --- | --- | --- | --- |
| QuickDASH – baseline | 64 (38.6 - 86.4) | 26 (0 – 65.9) | 0.000, t = -4.246  DOF 30 |
| QuickDASH – 3 Months | 33 (6.8 – 56.8) | 24 (0 – 54.5) | 0.283, t = -1.103  DOF 20 |
| QuickDASH – 12 Months | 28 (2.3 – 63.6) | 10 (0 – 25) | 0.035, t = -2.336  DOF 14 |

Supplementary Table 1. Pre- and post-operative patient reported QuickDASH score between groups across various time points.
