# Supplementary material for: ‘QuickDASH’ to find unique genes and biological processes associated with shoulder osteoarthritis: a prospective case–control study
Source: BMC Res Notes. 2024 Dec 19;17:361. doi: 10.1186/s13104-024-07035-9 (PMC11657115; doi:10.1186/s13104-024-07035-9)
Supplement: Supplementary file 16 — Supplementary material 16: Supplementary table 2. Baseline preoperative characteristics in the osteoarthritis and instability groups. All data rounded to 3 decimal places. Student’s t-tests were performed for continuous data. [file 13104_2024_7035_MOESM16_ESM.docx]

| Variable | Osteoarthritis  mean (range) | Instability  (mean, range) | p value |
| --- | --- | --- | --- |
| N | 6 | 26 |  |
| Sex (female) | 5 (83%) | 5 (19%) | 0.006 Fisher’s exact  0.002 Pearson chi2 |
| Age (years) | 71 (59-81) | 24 | 0.000 (t stat -18.455, 30 DOF) |
| BMI (kg/m^2^) | 31 | 26 | 0.0157 (t stat -2.566 29 DOF |
| Current Smoker | 0 | 8 (31%) | 0.298 Fisher’s exact test, 0.108 Pearson chi2 |
| GFR (ml/min/1.73m^2^) | 74 | 88 | 0.000 (t stat 3.927, 29 DOF) |
| CRP (mg/L) | 4.8 | 3.2 | 0.0407 (t stat -2.150, 27 DOF) |
| Urea (mmol/L) | 6.2 | 5.1 | 0.090 (t stat -1.752, 29 DOF) |
| HbA1c (%) | 5.7 | 5.0 | 0.000 (t stat -4.302, 29 DOF) |
| Fasting glucose (mmol/L) | 5.65 | 4.64 | 0.0001 (t stat -5.475, 13 DOF) |
| ASA | 2 | 1 | 0.001 (t stat -3.869, 29 DOF) |
| Hypercholesterolaemia | 5 (83%) | 0 | 0.000 Fisher’s exact test, 0.000 Pearson chi2 |
| Hypertension | 4 (67%) | 0 | 0.000 Fisher’s exact test, 0.000 Pearson chi2 |
| Abnormal thyroid function | 0 | 4 (15%) | 0.561 Fisher’s exact test, 0.294 chi2 |
| Abnormal LFTs | 3 (50%) | 4 (15%) | 0.110 Fisher’s exact test, 0.074 chi2 |
| Comparing patients undergoing surgery for osteoarthritis with surgery for instability; t-tests for continuous data | | | |

Supplementary Table 2. Baseline preoperative characteristics in the osteoarthritis and instability groups. All data is rounded to 3 decimal places.
