# Supplementary material for: ‘QuickDASH’ to find unique genes and biological processes associated with shoulder osteoarthritis: a prospective case–control study
Source: BMC Res Notes. 2024 Dec 19;17:361. doi: 10.1186/s13104-024-07035-9 (PMC11657115; doi:10.1186/s13104-024-07035-9)
Supplement: Supplementary file 17 — Supplementary material 17: Supplementary table 3. Top genes correlating with QuickDASH according to periarticular tissue with minimal significant fold change. All data rounded to 3 decimal places. [file 13104_2024_7035_MOESM17_ESM.docx]

Supplementary table 3. Top genes correlating with QuickDASH according to periarticular tissue with minimal significant fold change. All data rounded to 3 decimal places

| **Gene ID** | **Gene Rank** |
| --- | --- |
| C1QC | -9460 |
| C1QB | -9444 |
| MASP1 | -9418 |
| IGKV3-11 | -9413 |
| IGHV1-2 | -9408 |
| C1QA | -9396 |
| IGHV3-30 | -9344 |
| IGLV2-14 | -9330 |
| IGKV1-5 | -9324 |
| IGLC2 | -9238 |
| IGLV3-25 | -9227 |
| IGLV3-21 | -9180 |
| IGHV3-23 | -9173 |
| IGKV4-1 | -9151 |
| IGLV1-47 | -9148 |
| IGLV1-40 | -9126 |
| C1R | -9125 |
| IGLV2-11 | -9099 |
| IGHG2 | -9096 |
| IGKV3-20 | -9063 |
