# Supplementary material for: ‘QuickDASH’ to find unique genes and biological processes associated with shoulder osteoarthritis: a prospective case–control study
Source: BMC Res Notes. 2024 Dec 19;17:361. doi: 10.1186/s13104-024-07035-9 (PMC11657115; doi:10.1186/s13104-024-07035-9)
Supplement: Supplementary file 18 — Supplementary material 18: Supplementary table 4. Top 20 genes, creation of C4 and C2 activators. The Gene Rank is based on the rank of the DESeq2 test statistic value, centred around zero which determines no change in expression. [file 13104_2024_7035_MOESM18_ESM.docx]

| **Gene ID** | **Gene Rank** |
| --- | --- |
| C1QC | -9460 |
| C1QB | -9444 |
| MASP1 | -9418 |
| IGKV3-11 | -9413 |
| IGHV1-2 | -9408 |
| C1QA | -9396 |
| IGHV3-30 | -9344 |
| IGLV2-14 | -9330 |
| IGKV1-5 | -9324 |
| IGLC2 | -9238 |
| IGLV3-25 | -9227 |
| IGLV3-21 | -9180 |
| IGHV3-23 | -9173 |
| IGKV4-1 | -9151 |
| IGLV1-47 | -9148 |
| IGLV1-40 | -9126 |
| C1R | -9125 |
| IGLV2-11 | -9099 |
| IGHG2 | -9096 |
| IGKV3-20 | -9063 |
